# Psychological stressors of imprisonment and coping of older incarcerated persons: a qualitative interview study

**DOI:** 10.1186/s12889-025-21452-w

**Published:** 2025-01-27

**Authors:** Stuart McLennan, Leila Meyer, Tenzin Wangmo, Jens Gaab, Bernice Elger, Helene Seaward

**Affiliations:** 1 Institute for Biomedical Ethics, University of Basel, Bernoullistrasse 28, 4056 Basel, Switzerland; 2 Institute of History and Ethics in Medicine, Department of Preclinical Medicine, TUM School of Medicine and Health, Technical University of Munich, Munich, Germany; 3 Division of Clinical Psychology and Psychotherapy, Faculty of Psychology, University of Basel, Basel, Switzerland; 4 Center for Legal Medicine (CURML), Medical Faculty, University of Geneva, Geneva, Switzerland

**Keywords:** Prisons, Psychological Stressor, Coping Skills, Psychological Well-Being, Elderly persons in prison, Older persons in prison

## Abstract

**Background:**

Imprisonment has a major impact on a person’s psychological well-being. The proportion of older imprisoned persons is dramatically increasing worldwide, and they are likely to have greater physical and mental health needs compared to younger persons in prisons. However, there is currently a lack of research on the psychological stressors and the coping strategies of older imprisoned persons. This study therefore aims to explore the key psychological stressors experienced by older imprisoned persons and their coping strategies.

**Methods:**

Individual semi-structured qualitative interviews were conducted between April 2017 and December 2018 with a purposive sample of 79 participants from 2 different groups in Switzerland: older imprisoned persons (*n* = 50) and mental health professionals (*n* = 29) with experience working in prisons. Transcripts were analysed using conventional content analysis.

**Results:**

This study has identified various ways in which the prison environment not only undermines older incarcerated persons´ psychological well-being, but also their ability to manage the stress they are experiencing. Two overarching psychological stressors identified were a lack of physical and emotional closeness in social relationships (with prison staff, with other incarcerated persons, and outside of prison), and the loss of autonomy in prison. Participants reported five main ways that older incarcerated persons coped with the psychological stressors in prison: 1) recognising a lack of control over situation, 2) withdrawing and isolating, 3) self-improvement, 4) staying connected to the outside world, and 5) self-expression.

**Conclusions:**

To improve the psychological well-being of older incarcerated persons, there is a need for: specialised training of prison staff regarding hardships that elderly persons can face in prison relationships, encouragement and enablement of social contacts with the outside world, and increasing possibilities for autonomy, new challenges, and thus stimuli within a limited setting.

**Supplementary Information:**

The online version contains supplementary material available at 10.1186/s12889-025-21452-w.

## Background

Psychological stress has been defined as “a particular relationship between the person and environment that is appraised by the person as taxing or exceeding his or her resources or endangering his or her well-being” [1]. Existing research has found that key psychological stressors of imprisonment include being separated from loved ones, the absence of freedom and specific activities and objects, isolation, and the presence of social conflicts with other incarcerated persons or prison staff [2–9]. The term coping describes ways in which humans respond to said psychological stress, i.e. the perception of either personal threat, harm or loss, in efforts of prevention or at least reduction of its impact [10]. Psychological stressors and coping is a well-investigated subject in psychological research, including in the prison context [1, 7, 10]. However, research examining what are the psychological stressors and coping mechanisms of older incarcerated persons remains limited [11, 12].

The limited existing research with older incarcerated people identifies increased physical and mental health needs, mobility, social care needs, and victimisation, as the most prominent psychological stressors [13]. Other psychological stressors specific to older incarcerated persons include the possibility of dying in prison, and the worries of leaving behind same age partners [14–16]. It has also been found that some of the coping strategies used by older imprisoned persons evolved around religion and spirituality, but also “reminiscing, the recalling of happy memories and/or challenging past events successfully dealt with.” [11]. There is also a risk of inadequate medication or overprescribing of hypnotics and anti-anxiety drugs as a reaction to multiple stressors in prison [17–25].

Gaining better insight into the psychological stressors and coping strategies of the elderly incarcerated persons is important given the proportion of older imprisoned persons is dramatically increasing worldwide [26–30]. Furthermore, older incarcerated persons experience more somatic and mental health issues than same-age counterparts in the general community [31, 32]. Indeed, one study found that elderly incarcerated persons’ prevalence for psychiatric disorders was more than twice as high than that of older people in the general community [11]. Finally, the needs and challenges related to elderly incarcerated persons´ healthcare differ from the younger prison population [33–35]. At present, these needs are often not sufficiently met by institutions [11, 15, 36], who typically implement a “one‐size‐fits‐all regime” [11]. The study therefore aims to explore the psychological stressors and coping strategies of elderly incarcerated persons in Switzerland. Switzerland provides an interesting context to examine this issue given the proportion of imprisoned persons aged 49 years or older has almost tripled between 1984 to 2019, going from 6,6% to 17.7% [37].

## Methods

The methods of the study are presented in accordance with the “Consolidated criteria for reporting qualitative research” (COREQ) [38].

### Research team and reflexivity

#### Personal characteristics

Interviews were conducted by two female PhD students in biomedical ethics, who received training in qualitative research and supervision during data collection.

#### Relationship with participants

No relationship was established between the interviewers and the participants prior to the study and participants received limited information about the interviewers. There was no hierarchical relationship between the interviewers and the study participants.

### Study design

#### Theoretical framework

The theoretical framework employed in this study was conventional content analysis [39]. Conventional content analysis is generally used with a study design whose aim is to describe a phenomenon, in this case the views and experiences of participants regarding the psychological stressors and coping strategies of elderly incarcerated persons in Switzerland.

#### Participant selection

Participants were selected through purposive sampling [40] to ensure that participants were from different facilities and regions to increase the representativeness of findings. Participants were recruited from two groups. First, older incarcerated persons in Switzerland who were aged 50 years and above and had been in contact with mental health services at least once. We contacted all institutions housing adults sentenced to long-term imprisonment in the French- and German-speaking regions of Switzerland, which represent the country's two major language areas. The Italian-speaking region (one prison) was excluded due to feasibility constraints. Additionally, we excluded institutions housing juvenile or remand prisoners, as well as administrative detention centers that accommodate migrants awaiting deportation, due to the low number of older adults and the absence of long-term sentences in these facilities. Fourteen prisons and forensic-psychiatric institutions with medium to high security standards participated. While the Swiss Criminal Code (SCC) regulates penal law nationally, ensuring uniformity in sanctions imposed for specific crimes, implementation of sentences is governed at the cantonal level. Each canton determines the precise execution of sentences, resulting in variations in aspects such as the placement and management of mentally ill individuals [41]. Given the anticipated differences between institutions, we ensured data collection from a wide range of facilities and regions to increase the representativeness of our findings.

The incarcerated persons were made aware of the study by either the prison administration or mental health services. Study information and informed consent forms were handed out for distribution to potential participants. The study purpose, data confidentiality as well as the possibility of refusal at any time point were also verbally explained to participants. Written informed consent was then obtained from participants. Second, mental health professionals (such as psychiatry, psychology, and psychiatric nursing) who had a minimum of 10 years work experience in prisons, and who were based in the German or French speaking part of Switzerland. All mental health professionals had some experience in working with older adults, but none were specialised in geriatric psychiatry. Professionals were contacted directly about their willingness to participate by telephone or email. Informed consent forms were sent directly to the experts and consent was obtained on the day of the interview.

Setting: Interviews with older incarcerated persons were held between December 2017 and December 2018 at the institution the person was incarcerated. Interviews with mental health professionals were held between April 2017 and January 2018 either in person at a location of the participants choosing or via telephone or video call. Interviews were conducted in whichever language (either French, English, German, or Swiss German) the participant felt most comfortable with. Only the participant and the researcher were present during the interview. Data collection: Researcher-developed semi-structured interview guides were developed to guide the discussion (see Additional file 1, which includes Fig. 1). Interviews with older incarcerated persons explored: 1) personal circumstances in the imprisonment context: description of a typical day, social network, 2) mental health and care in prison: accessibility, treatment satisfaction, perceived stigma, and 3) aging in imprisonment: experiences, relationships to younger imprisoned persons, work and free time activities, perception of suitability of the environment, future plans during and after imprisonment. Interviews with professionals explored: 1) motivation and work experience, 2) organization of mental health care in their institution: treatment characteristics, opinions on current accessibility, patient motivation, 3) older patients: experiences, characteristics of care and interaction, similarities and differences of care in younger and older patients, 4) role conflict: “dual loyalty” (patient versus system), description of collaboration with other professions and representatives of the justice system, and 5) risk assessment and reporting to the authorities: characteristics, procedures, age as a variable in risk assessments, key criteria in reporting standards, examples. Based on the first two interviews that did not show any problems, it was decided that no further piloting or adaptation of the interview guides was necessary. No repeat interviews were carried out. Interviews were audio recorded and field notes were taken after the interviews. Interviews with older incarcerated persons lasted an average of 69 min (range 16–120 min), and interviews with professionals lasted an average of 71 min (range 48–90 min). The interviews were transcribed in the respective language, except for Swiss German interviews which were transcribed in Standard German as is common practice in the German-speaking area of Switzerland. Transcriptions were subsequently checked for accuracy and quality, and potentially identifying information removed. Data saturation was reached when no new information could be obtained, no additional codes emerged, and sufficient data was available to replicate the study [42]. We conducted data analysis concurrently with ongoing data collection, allowing for the inclusion of additional participants if necessary. We continued sampling until 50 interviews for older incarcerated adults and 29 for the mental health professionals to reach meaning saturation [43]. We explain this high number of participants to the inclusion of individuals from two distinct language regions and differences between institutions of the prison systems, such as how they specifically address aging in prisons or provide access to mental health care.

Transcriptions of the interviews were returned to participants on request, however, no corrections were received.

### Analysis and findings

Using the interview transcriptions in their original language, conventional content analysis was performed with the assistance of the qualitative software MAXQDA. Analysis commenced after interviews were completed by LM. Initial themes identified common across participants as well as those unique to individuals were labelled using a process of open coding. Findings are presented as higher- and lower-level themes. The other investigators [SM, TW, JG, BE, HS] reviewed the initial analysis to clarify and refine codes, and conversations among the investigators continued until coding differences were resolved and consensus was achieved. Selected quotes presented in this manuscript were translated into English and checked to ensure that the meanings were captured properly by the researchers fluent in German, French, and English.

## Results

A total of 79 participants agreed to participate in the study, including 50 older incarcerated persons, and 29 mental health professionals (see Table 1). Overall, 80% (63/79) of participants were male presenting, and 56% (45/79) of participants were from German-speaking areas in Switzerland.

**Table 1:** Participant characteristics

Characteristic		Older Incarcerated Persons *N* = 50	Mental Health Professionals *N* = 29	Total *N* = 79
**Gender** **n (%)**	Female	8(16)	8(28)	16(20)
	Male	42(84)	21(72)	63(80)
**Language Region** **n (%)**	French	21(42)	13(45)	34(43)
	German	29(58)	16(55)	45(56)
**Age**	Average	60	N/A	N/A
	Range	50 – 76		
	Standard Deviation	7.34		

## Psychological stressors

An overarching psychological stressor that was consistently reported by both participant groups was a lack of physical and emotional closeness for older incarcerated persons in social relationships.

### With Prison Staff

Mental health professionals reported that it can be challenging for prison staff to determine how much emotional or physical proximity they can and want to allow with incarcerated persons, given their primary duty is not to build relationships with the imprisoned persons but to perform an official act of the justice system:

*“The basic attitude of the supervisors is not to touch people…it's a pretty divided world, keeping your distance and also not becoming too personal in the exchange.” (Mental health professional_001)*

*“And the lack of interpersonal relationships. That is different in prison. The prison staffs’ job is not to have relationships with the prisoners but supervise them.” (Mental health profesional_002)*

### With other incarcerated persons

Participants also reported a lack of emotional warmth in the interactions among the imprisoned persons themselves. Group dynamics in prison were described as harsh. Older incarcerated persons referred to the importance of not interfering with others, but also a need to mark one´s presence at the same time:

*“Yeah, yeah, it's essential to be very careful, not to meddle in things here, but it’s essential also not to let yourself be impressed, to show you're here. You know it's all. It’s a little like in the animal kingdom. We have to show our place, our rank. You see, our territory, to mark our territory.” (Older incarcerated person_002)*

Both groups of participants reported that this dynamic made it difficult for older incarcerated persons to judge who they can trust and open up to. Indeed, many older incarcerated participants said that they found it difficult or had even completely stopped opening up to other imprisoned persons, feeling they could not show vulnerability in this setting as this would lead to them either be not understood or taken advantages of. This situation led to feelings of isolation and loneliness:

*“The sense of isolation and loneliness in a prison, it is colossal and in in my opinion it is completely underestimated…although there is lots of people, you do spend a lot of time on your own. You spend a lot of time on your own physically but more importantly mentally.” (Older incarcerated person_003)*

### With social contacts outside of prison

Another key psychological stressor for older incarcerated persons in particular were difficulties maintaining social contacts outside of prison. Three key barriers were identified:

- *Aging of friends and family:* It was reported that older incarcerated person´s social network became smaller as their family and friends increasingly died or their health and mobility deteriorated meaning they were no longer able to visit: *“Well, I don't have an extraordinary, inexhaustible network, because people start dying, you know.” (Older incarcerated person _002)*
- *Feeling of being a burden:* Some older incarcerated persons also felt like a burden to family and friends. This led to some to not be willing to discuss with their visiting children about issues they were experiencing inside prison to protect the parent–child relationship, requiring them to find other ways of dealing with their issues. Other older incarcerated persons reported that they understood that their friends and family may not want to stay connected anymore or had already broken off contact.
- *Institutional regulations:* Another barrier for maintaining social contacts frequently reported by participants was institutional regulations that were often unsupportive of outsides contacts and sometimes actively impeded them. Indeed, some older incarcerated person thought that institutions failed in their duties of enabling imprisoned persons to keep a connection to the outside world.
- Loss of autonomy

Another overarching psychological stressor was the loss of autonomy in prison. Older incarcerated persons reported struggling with the complete lack of choice they have in prison in terms of their movement, what they eat and wear, and what to do with themselves:

*“That is basically your free movement, your free decision what to do with yourself in here, that is completely taken away from you. You are completely ordered from morning to night what you have to do.” (Older incarcerated person _014)*

Partly because of this limited choice, older incarcerated persons also reported struggling with the loss of self-responsibility, and how this was a major barrier to successful integration following release from prison:

*“Here the food is put on the table and you have zero personal responsibility in here, zero, nada. I don't even have to think about the appointment with you. […] Here you are told everything (emphasised). […] All responsibility is taken away from you here. […] And I also believe that this is simply not going to lead to success if you want to live well and without offence afterwards.” (Older incarcerated person _429)*

*“The biggest challenge in my situation is getting back into everyday life and being able to manage my everyday life […in prison] we're a bit like babies, old babies and then, well, if I find myself like that in my own in a flat, well I'll have to relearn how to manage, I'll have to relearn how to get back into the flow of things myself, and that's a challenge!” (Older incarcerated person_443)*

*“We're being infantilised! They make us IDIOTS, they make us deliberately stupid! And I can't stand it!” (Older incarcerated person _445)*

Furthermore, older incarcerated persons reported that this lack of autonomy could be exacerbated by a lack of engagement with prisoners, for instance, regarding undiscussed changes in the prison environment:

*“Well, the first thing I would say is there would be […] more engagement with the prisoners. There would be some sense of dialogue with them. So, for example, if they are going to change something here. For example, they changed what you could purchase from the canteen recently. There was no conversation as to what people may actually want. They just said "here is the new list". "Thank you very much". People have written a monthly letter saying: "we would like this", the answer is no. Every time the answer is no. There is, there is a complete lack of engagement.” (Older incarcerated person _412)*

Older incarcerated persons also reported that a lack of new challenges in the limiting incarceration setting was particularly stressful, and that attempts to create new challenges in the incarceration setting were often frustrated by a lack of access to information due to the institution’s restrictions and that their suggestions for changes would usually be ignored. These left many older incarcerated persons with a sense of resignation:

*“I have gone through many attempts at different projects at some stages, but they are very difficult to fulfil because of the complete lack of access to information. (…) but realistically that is about it. Because everything else I have come across just results in frustration and disappointment. (Older incarcerated person_412)*

## Coping strategies

Participants reported five main ways that older incarcerated persons coped with the psychological stressors they experienced in prison:

One coping strategy many older incarcerated persons used was recognising the lack of control they had over the situation. For some, this took the form of actively accepting and acknowledging the reality of their situation:

*“Uh, I just take many things as for granted and say radical acceptance because everything else is [pointless] isn’t it. It has no value at all, it's no use, it's just the way it is, isn’t it.”* (Older incarcerated person_403)

However, others experienced resignation, and gave up to avoid frustration and disappointment:

*“So you give up and you continually give up. And it happens to everybody who comes here. You are trying, you are trying, you are trying, and you just give up to the point whereby you do not start to avoid frustration, disappointment, and everything yeah. It is the self-fulfilling prophecy so why begin?”* (Older incarcerated person_412)

### Withdrawing and isolating oneself

Some older incarcerated participants reported actively withdrawing and isolating themselves to cope with the situation. They withdrew emotionally, becoming cold and not showing emotion or opening up to others so they would not be exploited:

*“Actually prison taught me, you have to become cold, psychologically cold. Right, you can cry in your cell when you're locked up. But as soon as you exit the cell door, you are no longer allowed to show this side, because it is shamelessly exploited, isn't it.” (Older incarcerated person_005)*

*“But there are also moments…when I, or where I would feel like confiding in someone or where you feel like letting your feelings in the heart run free. But you can't. Yes, you can, you could go to someone and talk their head off, but it doesn't help you. Because you are not understood. And that's why I've become very introverted, or even more so now.” (Older incarcerated person_007)*

Others physically retreated to their own space to protect themselves from assault or making mistakes in social contacts with others:

*“Withdrawing protects you from assault. Withdrawing protects you from such machinations. Withdrawing protects you from an offer to gamble for money. Withdrawing protects you. That's what I've learnt. Um, withdrawing protects you from many mistakes you can make. In social contact. In interpersonal relationships. And perhaps waiting a bit more to simply be asked to be part of something. But then there's the "Have you got any money? I need it, can you lend it to me" situations, where you simply have to answer negatively. Withdrawing can save you from a lot of things that shouldn't have happened. […] That is indeed the case and you can protect yourself to a certain extent from unpleasant encounters. Nevertheless, you shouldn't do it too much, you have to be a bit socially acceptable and show social skills and of course you shouldn't withdraw permanently. […] So that's the advice I would give. ‘Withdraw more.’ I would then give him the example and say, ‘He's done everything right, he's out now. He came six months after me and is leaving a year before me. That's an extra year and a half that I'm sitting here just because this, that and the other thing happened to me. That shouldn't happen to you. Make sure it doesn't happen to you. Pull back more.’ ” (Older incarcerated person_425)*

### Self-improvement

Many older incarcerated persons felt that the limiting environment in prison could be a good opportunity to improve themselves. Some used their incarceration to find spiritual peace and take a new positive path:

*“Great peace, great joy. It's absurd to say, but it's a great joy, a great peace, and an absolutely much more sincere spiritual life, I've never experienced as much peace as in prison…I feel completely at peace, both with God and with people…I feel all clean and I can envisage one day going to heaven. Before I had a life that wasn't clean, now it is. So, for me, prison, I could also say it was something very positive to put me back…to prepare me for what's essential…well that's the path I've chosen, the Christian path.” (Older incarcerated person_409)*

Many also saw their incarceration as a chance to work on their personal issues and grow as an individual; with some highlighting the opportunity for younger incarcerated persons to learn from older incarcerated persons:

*“These are the chances you have in here. So you have to be in here, but you also have a chance inside, namely, that you can learn something. [I would tell them] That they make use of the opportunities. And be it on an interpersonal level, precisely from older people (...) use that and also learn it.” (Older incarcerated person_004)*

### Staying connected to outside world

Some older incarcerated persons reported consuming as much of the outside world as possible via different media and social contacts as a way of staying in touch with what was happening outside of prison, so that they would not be completely overwhelmed with the outside world once their incarceration ended:

*“For when I'll be out, I won't do: ‘Oh, I'm completely lost! What is that?’. You see, to have contacts on the outside, continue to stay busy, listen to broadcasts, read. I receive a newspaper every day here, so I read everything that's going on in the world. You see. I can stay/ it’s not because I'm here that I have to stay completely closed off. I can try to be myself.” (Older incarcerated person _002)*

### Self-expression

Another way older incarcerated persons coped with their stressful situation was through self-expression. Some reported using writing to express their feelings and to work through issues:

*“Some of this is work I did myself… I went through a very deliberate exercise of writing what I was thinking and I put it in a very particular structure, hum not for anybody else to read but writing for the sake of writing, it is a way of expressing it.” (Older incarcerated person_412)*

Others spoke of maintaining dignity through the way they presented themselves:

*“I can try to maintain a certain dignity, not let myself go. That's the most important thing. I think. Just because we're here doesn't mean that we let ourselves go, don't dress well, don't wash well, don't feel good, don't feel good, don't feel good, don't feel good, don't feel good, don't feel good, don't feel good, don't feel good….And if you start complaining too much, that's also a way of letting yourself go.” (Older incarcerated person_431)*

## Discussion

This is the first study to examine the issue of psychological stressors and coping strategies of the elderly incarcerated persons in Swiss prisons and makes an important contribution to the limited research on this topic internationally. This study has identified various ways in which the prison environment not only undermines older incarcerated persons´ psychological well-being, but also their ability to manage the stress they are experiencing. This often reflects persistent structural issues such as multiple stressors in prison and limited resources [17] that many other countries also face and has important implications for the successful reintegration of elderly prisoners into society after imprisonment.

The challenges identified regarding the lack of physical and emotional closeness in relationships with others were prominently discussed by participants and are generally in line with the existing literature. With regards to interactions with prison staff, the “tension between custody and care” that leads to the distance between prison staff and incarcerated people has been previously discussed [44–46]. Humblet summarises the dilemma as follows: “Prison staff have the daunting task of adopting a more custodial role (prison officers) or caretaking role (prison nurses), and managing prisoners and their emotions, whilst also being discouraged from developing a genuinely close relationship with them.” [44]. However, this emotional and physical distance of prison staff can lead to older incarcerated persons feeling the need to deal with negative emotions on their own. This is problematic, as the perception of caring or uncaring behaviour of prison staff has an important influence on well-being and coping in prison [46].

In relation to interactions with other incarcerated persons, due to harsh group dynamics and the lack of trustworthiness among the imprisoned persons, older incarcerated persons were very hesitant to self-disclose because of previous experiences of being exploited after doing so. This is problematic as it leads to imprisoned persons to actively withdraw, leading to further isolation. This is consistent with previous research in Australia that found that a lack of supportive prison relationships was related to isolation and loneliness in older incarcerated persons [12]. Aging incarcerated persons in the United States have also reported similar struggles regarding emotional closeness, with many participants having “topics they would never discuss with other inmates” [47]. Multiple other studies have also identified themes of trustworthiness in interactions with other imprisoned persons as a general cause for psychological stress [47–50].

Concerning social contacts outside of prison, older incarcerated persons found their slowly diminishing social networks because of aging peers and family members particularly stressful. Dealing with separation or even death of close peers or family have been identified as a significant psychological stressor by previous studies [15, 51, 52]. This situation was reported to be exacerbated by institutions’ strict regulations regarding visits etc. A possible cause for this could lie in the ongoing dilemma of the justice system prioritising public safety concerns over holding connections to the outside world [47]. In our study, persons already in incarceration for a long period of time reported security regulations becoming increasingly stricter, which is in line with previous research that found that older incarcerated participants without parole experienced rules on visits becoming more and more restrictive [53]. This lack of encouragement by institutions is an important barrier as it further isolates older incarcerated persons [54], and calls into questions the willingness of the state and its institutions to support the resocialisation of incarcerated persons. Keeping connection to the outside world has been found to be crucial [55]; support from family members and peers acts as a positive predictor for both successful institutional adjustment [54] and reintegration [56]. Furthermore, it has been found that older incarcerated persons without such networks are often very anxious about coping with life after imprisonment [16, 52]. Older incarcerated participants feeling like a burden to outside social contacts is also mirrored by previous studies [54, 57]. These perceptions have been summarised as: “Inmates soon realize that family and friends on the outside cannot place their lives on hold for the duration of the inmates’ sentences, and thus, neither wish nor request their loved ones make personal sacrifices or to be burdened by them” [54]. This is problematic as it might lead older incarcerated persons to actively withdraw from outside social contacts, whose support is a great resource for coping with psychological stressors of imprisonment [58].

Apart from the factual deprivation of autonomy through being confined, older incarcerated persons in our study also spoke about struggling with their limited autonomy within the setting, and also a lack of new experiences and thus stimuli leading to perceptions of monotony and boredom in imprisonment, which is an issue commonly reported by previous research [6, 9, 17, 51, 59]. This is concerning as perceptions of boredom and lack of control predict how well an incarcerated person can adjust emotionally to imprisonment [6, 9, 60]. Indeed, older incarcerated persons’ responses revolving around the subject of resignation are particularly concerning, as they imply a lacking ability to cope with the concerned psychological stressor [9].

Continuous exposure to stress has been shown to strain the cardiovascular and immune systems in the general population, leading to an increased risk of health issues [61, 62]. Imprisonment has been characterized as a stressful experience and incarcerated persons must manage these challenge [62]. Certain coping strategies can be classified as either healthy or unhealthy [63, 64], for instance, the use of adaptive versus maladaptive coping strategies is associated with varying levels of psychological symptoms, adjustment, and overall well-being [65, 66]. For this reason, the promotion of using healthy and adaptive coping strategies such as problem-solving, seeking social support, or engaging in physical activity is important due to its link between stress and health [67], particularly in view of the high prevalence of mental and physical illnesses in older incarcerated adults [68–70].

One of the earliest distinctions of coping approaches is between a problem-focused versus emotion-focused coping approach. A problem-focused approach aims at the factor causing the psychological distress and tackling it directly by removal, evasion or diminution, e.g. by either ending or working on a troublesome relationship, while an emotion-focused approach aims at reducing distress by emotion modulating, e.g. by displaying negative emotions (such as crying), looking for emotional support [10]. The major issue with the deprivation from closeness in social relationships in prison is that it can affect an incarcerated persons’ emotion-focused coping. In an environment where the individual feels they have no one to turn to for self-disclosure and comfort, seeking emotional support from others to deal with negative emotions is not possible. Furthermore, being enclosed in a cell for a major amount of time can also impede problem-focused coping. For the incarcerated person wishing to disengage as a coping strategy, the only option regarding flight is that of a “mental flight” (e.g. avoidance, denial). Freedom of physical movement, for example deciding to go outside for a walk to deal with a current stressor, does not lie within their control anymore.

### Limitations

The interviews included in this study were completed in 2017–2018, and therefore may not reflect current practices and views. However, this study takes a cross-sectional approach and the insights gained regarding the psychological stressors experienced by older incarcerated persons and their coping strategies remain valuable. Our study focused on how these issues affect older adults and did not aim to provide a comparison with other age groups. However, many stressors identified may not be exclusive to older incarcerated people. Further research should uptake comparative approaches between the age groups to disentangle what is common and specific for the age groups. As all participants had received mental health treatment (at least one interaction with the MH care services) the findings are based on the population of incarcerated older adults with mental health issues. Also, a bias might exist toward the reporting of socially desirable attitudes [71], however, given our results that are rather critical of current practice, we believe that such a bias is limited. The study was only carried out with a purposive sample and in Switzerland, and thus, the findings are neither generalizable to all older persons nor for other countries. Nevertheless, many of the key issues are associated with aspects that are common in all countries (e.g., multiple stressors in prison and limited resources), these findings are likely to be of wider international interest and as our findings showed great similarities to findings previous research from other countries.

## Conclusion

In conclusion, the prison environment not only undermines older incarcerated persons´ psychological well-being, but also their ability to manage the stress they are experiencing. Our findings show that emotion-based coping strategies are very limited and problem-based coping strategies are not available in this setting. This may create a greater risk of older incarcerated people experiencing low psychological well-being. To improve the psychological well-being of older incarcerated persons, prisons must address the main stressors of imprisonment such as facilitate meaningful contact within the institution and with social network outside. Further, we support the recommendation to improve the training of prison staff on this age group of the prison population, particularly in supporting them in adopting adaptive coping strategies [44]. The lack of national training available for prison staff on how to effectively support older prisoners has also been identified in other countries, such as the UK [72]. Developing such training or improving existing training could ultimately benefit not only older incarcerated persons, but also help prison staff with navigating this dilemma. As having a supportive outside social network is one of the predictors for successful reintegration after imprisonment [73], further enabling and encouraging connections to the outside world is also crucial. In an environment often perceived as under stimulating, it is especially important that older incarcerated persons can still experience some autonomy, new challenges and stimulation.

## Supplementary Information

Supplementary Material 1.

## Data Availability

The dataset analyzed during the current study is not publicly available. Our analysis is based on qualitative interviews with mental health professionals working in secure settings and older persons living in detention. The individual privacy of our study participants would be compromised if we shared the whole transcripts publicly. However, we can share the parts of the transcripts relevant for this paper upon reasonable request. Please contact either tenzin.wangmo@unibas.ch or helene.seaward@unibas.ch.
