# Supplementary material for: Psychological stressors of imprisonment and coping of older incarcerated persons: a qualitative interview study
Source: BMC Public Health. 2025 Jan 27;25:328. doi: 10.1186/s12889-025-21452-w (PMC11770940; doi:10.1186/s12889-025-21452-w)
Supplement: Supplementary file 1 — Supplementary Material 1. [file 12889_2025_21452_MOESM1_ESM.pdf]

Additional file 1: Interview Guide

**Part of the interview guide / theme / technique**	**Interview questions**	**AIM: What should be dealt with**
**Introduction**	Could you describe a typical day here in [name of the institution]?	• Ice breaker<br>• Getting to know daily life structure in prison
**Social Network**	Are you close to anybody within the institution?<br>• Staff<br>• other inmates<br>Do you keep in touch with anybody outside of the institution?	• How are relationships with others within the institution perceived?<br>• Also less contact is usually available to the outside world – how is this perceived, did they lose relationships, do they keep them?
**Transition from introductory to main part**	What do you think about the mental health care services in [name of the institution]?	• Get their general (very brief) impression about how mental health services are, how they feel taken care of.<br>• The goal is to ease into questioning them about psychological disorders (since it's a sensitive topic) and the treatments they receive for that.
**Diagnoses**	What kind of mental health disorders do you have?<br>• How long have you had this disorder for?<br>• Since when have you received treatment for your disorder?	• What is the **subjective disease model** of the patient?
**Prison Mental Health Care Services**	When did you first get in touch with the mental health care service?<br>• How?<br>• Who?<br>• Why?	• Elaborate **access** to mental health care services.<br>• How do they approach the staff (through nurses, regular consultations by psychiatric services, security staff…)?<br>• How are **decisions** taken to provide access to certain healthcare resources?
**Treatments**	What **type** of treatments do you receive for your mental disorder?	• **Equivalence of care**

	• In the institution<br>• Before imprisonment<br>• Any differences?<br>• Frequency - how often?<br>• Which specialists did you meet?<br>• Duration of a session?	• Differences between mental health care inside and outside of prison.<br>• Any **continuity** given? (same therapist? Contact between prison and outside mental health care staff?)<br>• What is considered as treatment in the view of the patient – what isn't?
	Do you think that anything has **changed** about your mental health while being here?<br>• Do you feel any improvements or degradation about your mental health issues?<br>• What treatment helps/has helped you most?<br>• Are you satisfied with these treatments?<br>• What treatment/activity would you most preferably drop?<br>• Do you think that you're treated differently because of your age?	• Explore what participant views as most helpful about treatment received.<br>• What is seen as most useless?
	• Do you talk to anybody of the other inmates about being in touch with mental health services?<br>• Has anything changed since you contacted the mental health service/you entered the institution?	• Explore possible threat of stigma, mobbing, and exclusion.
	If you could change anything about the treatment you receive, what would it be?<br><br>What type of additional health care would you need?	• Possible **improvement options** that the offenders see<br>• Their perspective on the **quality** of MH care
	**Elicitation Technique** (see Figure1)<br>Where do you place the mental health professional?<br><br>Where do you place the person conducting the forensic psychiatric expertise?	• How do they relate to mental health professionals?<br>• The MHP's dual role, trust and therapeutic relationship are important aspects in mental health care – use elicitation technique to evoke these issues.

**Locus of Control**	What do you do yourself about the mental health issues that you have?<br><br>What influences the **progress** of your therapy?<br><br>Who influences the progress of your therapy?	• How much self-efficacy do they experience?<br>• What do they see as most important therapeutic effect?<br>• What is the most important facilitator?<br>• Sentencing limitation – **indeterminate** character of the sentence
**Risk Assessment**	How do you experience the forensic risk assessment procedures?<br><br>From your point of view, what influences the decision on your progression within the institution and the time of release?	• How do they experience the risk assessment process conducted by the psychiatrists?<br>• How do they perceive **indeterminate** character of the sentence?
**Aging**	What is it like to be here? What is it like to become older here?<br><br>What is **most challenging concern** for you in your life right now?<br>• Over your course of the stay in your institution, what has changed for you? Do you face different challenges now?<br>• Do you think that aging has an impact on your everyday life in the institution?<br><br>Is the **prison environment** adapted to people of your age and older?<br>• What would you improve or need?	• What are the **challenges** of the older offender population?<br>• The open question shall give room for them to relate to the aspect that is most important to them<br>• How does aging shape their prison experience?
	What **plans** do you have with your life?	• What do they **hope** for?<br>• What plans do they have for the future and how does the older age possibly affect this?<br>• Do they have any future plans for after imprisonment?

**Closing up**	How would you advise a younger inmate who asks you how to deal with mental illness?	Enter third person to try to elicit a meta-description of themselves and their life living with a mental disorder within prison.

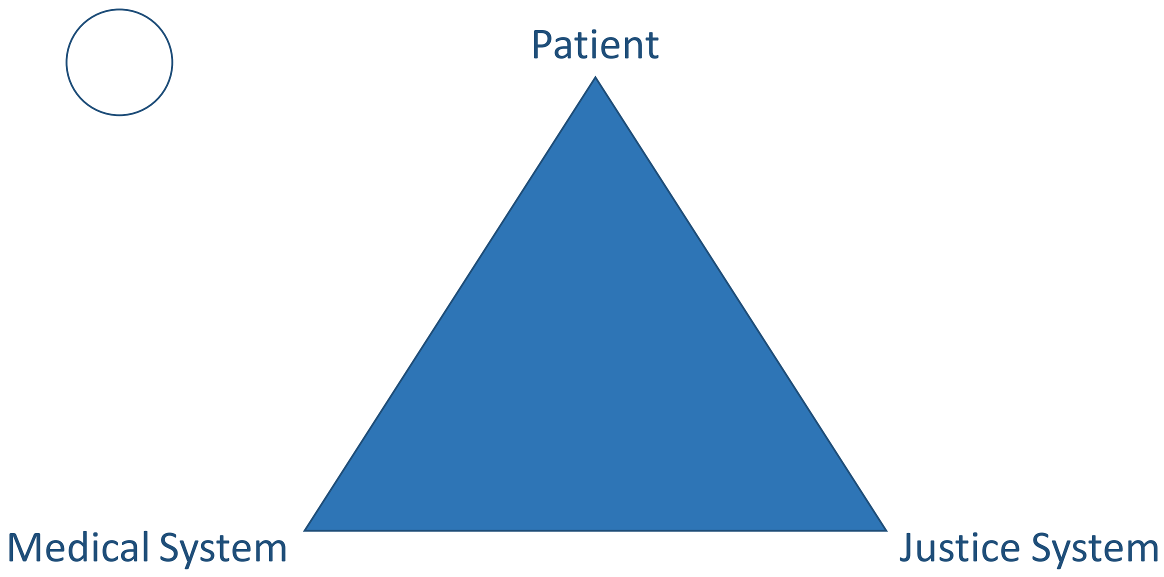

Figure1. Elicitation Technique
